# Ultrasound-Induced Cavitation as Biological Constraint Focusing: A Phenomenological Bioengineering Model for Sonoporation, Sonodynamic Therapy, Drug Delivery, and Histotripsy

**DOI:** 10.3390/bioengineering13070832

**Published:** 2026-07-21

**Authors:** Mădălina Duceac-Covrig, Călin Gheorghe Buzea, Florin Nedeff, Diana Mirilă, Valentin Nedeff, Mirela Panainte-Lehaduș, Claudia Manuela Tomozei, Maricel Agop, Daniela Andriuță, Carmen Laura Cristescu-Budală, Lăcrămioara Ochiuz, Decebal Vasincu

**Affiliations:** 1 Doctoral School of Biomedical Sciences, Faculty of Medicine and Pharmacy, Research Centre in the Medical-Pharmaceutical Field, “Dunărea de Jos” University of Galați, 47 Domnească Street, 800008 Galați, Romania; madalinaduceac@yahoo.ro; 2 Clinical Emergency Hospital “Prof. Dr. Nicolae Oblu” Iași, 700309 Iași, Romania; calinb2003@yahoo.com; 3 National Institute of Research and Development for Technical Physics—IFT Iași, 700050 Iași, Romania; 4 Department of Environmental Engineering, Mechanical Engineering and Agritourism, Faculty of Engineering, “Vasile Alecsandri” University of Bacău, 600115 Bacău, Romania; florin_nedeff@ub.ro (F.N.); vnedeff@ub.ro (V.N.); mirelap@ub.ro (M.P.-L.); claudia.tomozei@ub.ro (C.M.T.); m.agop@yahoo.com (M.A.); 5 Faculty of Physics, Alexandru Ioan Cuza University of Iași, 700506 Iași, Romania; dana.andriuta@gmail.com; 6 Faculty of Medicine and Pharmacy, Research Centre in the Medical-Pharmaceutical Field, “Dunărea de Jos” University of Galați, 47 Domnească Street, 800008 Galați, Romania; 7 Faculty of Medicine, “Grigore T. Popa” University of Medicine and Pharmacy Iași, 700115 Iași, Romania; lacramioara.ochiuz@umfiasi.ro (L.O.); decebal.vasincu@umfiasi.ro (D.V.)

**Keywords:** ultrasound-induced cavitation, therapeutic ultrasound, microbubbles, sonoporation, sonodynamic therapy, histotripsy, cavitation-mediated drug delivery, reactive oxygen species, biological constraint focusing, relational–informational model, bioactive relaxation, phenomenological bridge model

## Abstract

Ultrasound-induced cavitation is conventionally described through nonlinear bubble dynamics, acoustic pressure modulation, microbubble oscillation or collapse, local mechanical stress, thermal or chemical activation, and subsequent biological effects. In medical contexts, such cavitation-mediated processes are increasingly relevant to sonoporation, microbubble-enhanced drug delivery, sonodynamic therapy, and histotripsy. However, a compact phenomenological framework linking measurable cavitation dynamics to delayed, channel-specific biological outputs remains useful, particularly when different endpoints such as membrane permeabilization, reactive oxygen species generation, molecular uptake, and tissue fragmentation are considered together. In this work, a phenomenological relational–informational bridge model is proposed, in which therapeutic cavitation is interpreted as biological constraint focusing. The cavitation region and its adjacent biological microenvironment are represented as a localized, acoustically driven subsystem whose effective constraint state changes during bubble or microbubble oscillation and collapse. Bubble oscillation or collapse is represented as a rapid increase in constraint loading and informational action density, whereas medically relevant effects are modeled as relaxation of a transient high-tension state into bioactive output channels, including membrane permeabilization, reactive oxygen species generation, molecular delivery, and mechanical tissue fragmentation. The model couples the bubble or microbubble radius *R*(*t*) and collapse or oscillation velocity $\dot{R}(t)$, obtained experimentally or from Rayleigh–Plesset-type dynamics, to a dimensionless relational constraint parameter *λ*(*t*), an informational action density $S_{rel}(t)$, a stored high-tension reservoir $E_{rel}(t)$, channel-specific motif populations $N_{k}(t)$, and measurable biological outputs $B_{k}(t)$. The construction is not intended to replace hydrodynamic, thermodynamic, sonochemical, or biomechanical models; rather, it provides a latent-variable layer that may organize how cavitation loading is converted into endpoint-specific biological responses. The framework yields testable expectations: biological response should correlate not only with acoustic pressure or minimum bubble radius, but also with the rate of constraint loading, reservoir buildup and depletion, relaxation-channel kinetics, and modifiers such as microbubble composition, tissue context, oxygenation, sonosensitizer availability, and molecular cargo. Ultrasound-mediated cavitation is therefore reframed as a bioengineering process in which acoustic exposure, bubble dynamics, transient energy localization, and biological endpoint formation are connected through a testable phenomenological bridge model.

## 1. Introduction

Acoustic cavitation is one of the most striking examples of energy localization in nonlinear physical systems. In its purely physical form, an acoustically driven gas bubble suspended in a liquid may emit short flashes of light during violent collapse, a phenomenon known as sonoluminescence. In single-bubble sonoluminescence, a bubble trapped by a standing acoustic wave undergoes repeated expansion and collapse cycles, with light emission occurring near the final stage of collapse, when acoustic energy has been concentrated into an extremely small spatial and temporal region [1,2,3]. The phenomenon is remarkable because an apparently macroscopic mechanical drive—sound—can generate highly localized physical effects through a compressed, transient state of matter.

The same general class of cavitation-mediated processes is also relevant in medicine. In biomedical ultrasound, bubble oscillation and collapse may occur around endogenous gas nuclei, contrast microbubbles, or externally introduced cavitation agents. Depending on the exposure regime and biological context, such processes may contribute to membrane permeabilization, enhanced molecular transport, reactive oxygen species generation, tissue disruption, or mechanical ablation. These effects are central to several emerging or established ultrasound-mediated strategies, including sonoporation, microbubble-enhanced drug delivery, sonodynamic therapy, and histotripsy [4,5,6,7,8,9,10,11]. Thus, while sonoluminescence represents a physical radiative output of acoustic cavitation, medical ultrasound-mediated cavitation represents a broader class of bioactive outputs generated by the same underlying principle of localized acoustic energy concentration.

From a bioengineering perspective, an important challenge is that these cavitation-mediated effects are often measured through different endpoint-specific observables. Sonoporation may be quantified through membrane permeability or molecular uptake [4,5], sonodynamic therapy through reactive oxygen species generation or cell viability change [7,8,9], drug delivery through cargo accumulation [5,6], and histotripsy through tissue fractionation or lesion formation [10,11]. Although these outputs arise from related cavitation physics, their timing, magnitude, and dependence on microbubble composition, tissue context, oxygenation, and molecular agents are not always captured by a single descriptor such as acoustic pressure, mechanical index, or minimum bubble radius. This motivates a bridge model that separates cavitation loading, transient storage of a high-tension state, channel-specific relaxation, and measurable biological output.

The standard physical description of cavitation is built on nonlinear bubble dynamics, gas compression, thermal excitation, chemical activation, and, in the case of sonoluminescence, radiative emission. The bubble radius is usually treated as an effective dynamical variable governed by Rayleigh–Plesset-type equations, which describe the radial motion of a spherical cavity in a surrounding liquid under the action of acoustic pressure, surface tension, viscosity, and internal gas pressure [12,13]. During the rarefaction phase of the acoustic cycle, the bubble expands; during the compression phase, it may collapse rapidly. This collapse can produce extreme gas compression, high local temperature, possible ionization, plasma-like behavior, chemical excitation, and, in appropriate conditions, photon emission [1,2,3,14]. In biomedical settings, the same bubble dynamics may instead or additionally produce mechanical stress on membranes, transient permeability changes, local chemical activation, vascular or cellular effects, and tissue-level disruption.

This conventional framework has been highly successful in organizing much of the observed phenomenology. It explains why cavitation-mediated effects depend sensitively on acoustic pressure, gas species, liquid properties, ambient temperature, vapor content, bubble stability, and collapse dynamics [1,2,3]. In biomedical applications, the relevant parameter space is further expanded by microbubble composition, shell properties, tissue stiffness, perfusion, cellular membrane state, sonosensitizer availability, and local biological susceptibility. Nevertheless, both the physical and biomedical descriptions operate at the level of already-emergent variables: bubble radius, pressure, temperature, confinement, plasma state, membrane permeability, reactive chemical species, tissue disruption, and photon emission. These descriptions clarify how such variables interact, but they do not address whether they may themselves be effective manifestations of a deeper relational organization. The present study does not suggest that established cavitation theory fails to explain these phenomena. Rayleigh–Plesset modeling, microstreaming, shock-wave effects, sonochemistry, bubble-cloud dynamics, and membrane-poration models remain the primary mechanistic descriptions of cavitation-mediated bioeffects. The present model addresses a narrower problem: how cavitation loading may be represented as a compact latent state-space layer linking bubble dynamics to delayed, endpoint-specific biological outputs.

The relational–informational framework recently proposed by Buzea et al. offers a possible interpretive layer for such a bridge model [15]. In that framework, geometry, forces, spacetime, and particle-like excitations are interpreted as effective descriptions of constrained relational information. The fundamental structure is represented as a network of degrees of freedom linked by admissible informational relations, subject to constraints on accessibility or flow. Effective geometry emerges from minimal constraint cost, while effective forces arise as informational or entropic responses to gradients in constraint parameters [15]. Particle-like and excitation-like entities are likewise interpreted as stable localized motifs of constrained information flow. Within this ontology, a photon may be represented as a photon-like radiative relational motif, denoted by $H_{\gamma }$, whose coarse-grained manifestation is the ordinary electromagnetic photon γ. By extension, biomedical cavitation effects may be modeled as bioactive relaxation channels of a compressed relational configuration, without denying their standard mechanical, thermal, chemical, or biomechanical descriptions. The relational terminology used below is therefore not introduced ad hoc, but is adapted from the axiomatic relational–informational framework developed in Ref. [15]. In the present manuscript, however, this framework is used in a deliberately phenomenological and operational manner: it defines latent bridge variables linking standard cavitation dynamics to delayed, endpoint-specific biological outputs, rather than claiming a complete first-principles derivation of cavitation bioeffects.

In the present work, ultrasound-induced cavitation is modeled, at a phenomenological structural level, as biological constraint focusing. The collapsing or oscillating bubble is not regarded merely as a hydrodynamic cavity, but as an emergent boundary condition that dynamically compresses the accessibility structure of a localized relational subsystem. In medical settings, this subsystem includes not only the bubble interior, but also the adjacent biological microenvironment, including membranes, extracellular matrix, vascular structures, tissue interfaces, and molecular agents. The acoustic field acts as an external modulation of relational constraints; bubble oscillation or collapse corresponds to a rapid reduction in accessibility volume; gas heating, chemical activation, and mechanical stress are interpreted as coarse-grained proxies for informational action-density concentration; and the resulting biological effects correspond to relaxation of a high-tension relational configuration into bioactive channels.

The proposed interpretation does not discard the standard hydrodynamic, thermodynamic, chemical, biomechanical, or plasma-physical description. Rather, it treats these descriptions as effective macroscopic layers. Rayleigh–Plesset dynamics provides the observable evolution of the bubble boundary, while the relational–informational layer assigns this evolution a structural meaning in terms of constraint modulation, accessibility collapse, action-density amplification, motif destabilization, and relaxation-channel activation. In this sense, ultrasound-mediated cavitation becomes a medically relevant example of a more general principle: when relational accessibility is rapidly compressed by an external drive, local action density can rise sharply, generating a high-tension state whose relaxation may appear, after coarse-graining, as optical emission, chemical activation, membrane permeabilization, molecular delivery, or tissue fragmentation.

The aim of this paper is therefore to construct a bridge between nonlinear cavitation physics, biomedical ultrasound effects, and a relational–informational foundation of physical and biological description. A minimal phenomenological model is introduced in which the bubble or microbubble radius *R*(*t*), obtained from standard bubble dynamics, is coupled to a relational constraint parameter *λ*(*t*). This parameter controls accessibility compression and defines an informational action density $S_{rel}(t)$. To avoid identifying biological effect directly with compression alone, a stored high-tension reservoir *E_rel_*(*t*) is further introduced, whose relaxation forms a family of bioactive channel populations $N_{k}(t)$. The downstream expression of these channels determines measurable biological outputs $B_{k}(t)$, such as membrane permeability change, reactive oxygen species production, molecular uptake, or tissue fragmentation. In the relational ontology adopted here, photon emission remains one possible radiative channel, represented by $H_{\gamma }$, but medical cavitation is treated more generally as a family of bioactive relaxation pathways.

The contribution of the present work is not the invention of latent-variable, reservoir, or delayed-response modeling in general; such approaches are already well established in systems biology, pharmacokinetics, and dynamical modeling. Rather, the specific contribution is to formulate a cavitation-driven phenomenological bridge model in which the observable or simulated variables *R*(*t*) and $\dot{R}(t)$, obtained experimentally or from Rayleigh–Plesset-type dynamics, are mapped to reconstructed loading descriptors, a transient reservoir, channel-specific relaxation, and an endpoint functional *B_k_*(*t*). This functional can then be compared to conventional cavitation predictors such as acoustic pressure, *R_min_*, mechanical-index-like exposure descriptors, $\frac{\dot{R}}{R}$, and cavitation-dose metrics. The novelty is therefore the proposed organization of cavitation loading, transient storage, and delayed endpoint expression into a testable state-space framework for ultrasound-mediated bioeffects, while preserving ordinary hydrodynamics, thermodynamics, sonochemistry, biomechanics, and biomedical ultrasound theory as the effective descriptive layers.

## 2. Standard Physical and Biomedical Picture of Ultrasound-Induced Cavitation

Ultrasound-induced cavitation is conventionally understood as a nonlinear phenomenon in which acoustic energy is concentrated by the radial motion of a gas bubble or microbubble in a liquid or soft biological medium. In its classical sonoluminescent form, this process may culminate in a short optical flash near the moment of maximum collapse. In biomedical settings, the same general class of bubble dynamics may instead produce mechanical, chemical, permeability-related, or tissue-disruptive effects. In the standard view, the essential physical sequence is

**acoustic forcing** → **bubble or microbubble expansion** → **nonlinear oscillation or collapse** → **gas compression, mechanical stress, thermal or chemical activation** → **radiative or bioactive output.**

During the rarefaction phase of the acoustic cycle, the pressure in the surrounding medium decreases and the bubble expands. During the subsequent compression phase, the external pressure increases and the surrounding liquid or tissue-like medium accelerates inward, causing the bubble to collapse or undergo strong nonlinear oscillation. If the collapse is sufficiently rapid and symmetric, the gas inside the bubble is compressed into a very small volume over an extremely short time interval. This compression can produce high temperatures, large pressure gradients, shock-like behavior, chemical excitation, partial ionization, or plasma-like states, depending on the gas composition and liquid conditions [1,2,3]. In biological media, related cavitation dynamics may additionally generate local shear stress, membrane deformation, transient permeability changes, reactive chemical species, or mechanical tissue disruption.

The hydrodynamic core of this description is usually based on Rayleigh–Plesset-type bubble dynamics. For a spherical bubble of radius *R*(*t*) in a liquid of density ρ, viscosity μ, and surface tension σ, the radial motion can be written in simplified form as

(1) $$\rho \left(R\ddot{R}+\frac{3}{2}{\dot{R}}^{2}\right)=P_{gas}\left(R,t\right)-P_{\infty }\left(t\right)-\frac{2\sigma }{R}-\frac{4\mu \dot{R}}{R}$$

Here, $P_{gas}\left(R,t\right)$ is the pressure inside the bubble, while $P_{\infty }\left(t\right)$ is the pressure far from the bubble, including the externally applied acoustic pressure. The left-hand side represents the inertial response of the surrounding medium. The terms on the right-hand side represent, respectively, the internal gas pressure, the external driving pressure, surface tension, and viscous damping [12,13]. In biomedical applications, the effective values of these terms may be modified by microbubble shell properties, gas core composition, tissue viscosity, confinement, vascular boundaries, or nearby cellular and extracellular structures [6,16].

For therapeutic ultrasound, this hydrodynamic description represents only the first level of a broader bioengineering problem. The same bubble or microbubble trajectory that determines expansion, compression, and collapse also determines the mechanical, chemical, and interfacial loading transmitted to nearby membranes, extracellular matrix, vascular walls, or tissue interfaces. Thus, the measurable biological endpoint is not determined by acoustic pressure alone, but by the local coupling between acoustic exposure, bubble dynamics, microbubble properties, and the biological microenvironment.

In many treatments, the far-field pressure is expressed as

(2) $$P_{\infty }\left(t\right)=P_{0}-P_{a}\sin\left(\omega t\right)$$

where $P_{0}$ is the ambient pressure, $P_{a}$ is the acoustic pressure amplitude, and ω is the angular frequency of the sound field. This periodic forcing drives repeated expansion and collapse of the bubble. Stable single-bubble sonoluminescence occurs only within restricted parameter ranges, because the bubble must remain trapped, nearly spherical, and dynamically stable over many acoustic cycles [1,2,14]. In medical ultrasound, by contrast, the relevant regimes may include stable microbubble oscillation, inertial cavitation, cavitation-enhanced permeability, or destructive cavitation, depending on the acoustic exposure, microbubble properties, and biological context.

The thermodynamic part of the standard model concerns the gas inside the bubble. During slow expansion, the bubble can exchange heat and mass with the surrounding medium. During the final collapse, however, the compression may be so rapid that heat exchange becomes inefficient. Under such conditions, the gas temperature can rise sharply. A simplified adiabatic estimate gives

(3) $$P_{gas}V^{\gamma }\approx constant$$

or, equivalently, since $V\propto R^{3}$,

(4) $$T_{gas}(t)\sim T_{0}{\left(\frac{R_{0}}{R(t)}\right)}^{3(\gamma -1)}$$

where γ is the heat-capacity ratio of the gas. This expression is only an idealized approximation, because real sonoluminescing or cavitating bubbles involve vapor, heat conduction, molecular dissociation, chemical reactions, gas diffusion, and possible shock formation [1,2,3]. Nevertheless, it captures the central physical intuition: as *R*(*t*) becomes very small, compression can strongly amplify the internal temperature and local energy density.

In sonoluminescence, the emitted light is interpreted as the radiative signature of this extreme transient state. Several microscopic mechanisms have been discussed in the literature, including thermal bremsstrahlung, recombination radiation, molecular emission, blackbody-like emission from a hot dense core, and radiation associated with excited atomic or molecular states [1,2,3,17,18,19]. The relative importance of these mechanisms depends on the gas species, liquid composition, vapor content, collapse intensity, and degree of ionization. Thus, while the broad sequence of events is well established, the detailed microscopic origin of the emitted spectrum and pulse width remains a subtle problem.

In biomedical cavitation, the corresponding output need not be optical. Bubble oscillation or collapse may instead be associated with mechanical stress, membrane permeabilization, enhanced molecular transport, reactive oxygen species generation, drug or gene delivery, vascular effects, or tissue fragmentation [4,5,6,7,8,9,10,11]. In sonoporation and microbubble-enhanced delivery, cavitation activity can transiently increase membrane permeability and facilitate molecular or particulate uptake [4,5,20]. In sonodynamic therapy, cavitation-associated sonochemical processes may contribute to reactive oxygen species production, particularly in the presence of sonosensitizers and adequate oxygenation [7,8,9]. In histotripsy, intense cavitation activity can produce mechanical tissue fractionation through repeated bubble-cloud expansion and collapse [10,11]. These effects are not separate from the physical picture, but represent endpoint-specific biological expressions of cavitation dynamics under different acoustic, material, and tissue conditions. The relevant output is therefore not only emitted radiation, but a broader family of measurable biological responses.

For the purposes of the present paper, the important point is that ordinary physics describes cavitation through an effective hierarchy of variables and processes. The acoustic pressure field drives the bubble radius *R*(*t*); the collapse or nonlinear oscillation of *R*(*t*) compresses the gas and loads the surrounding medium; compression generates high-temperature, excited, chemically active, or mechanically stressed states; and these states relax through radiative, chemical, mechanical, or biological pathways. This effective description is powerful and must be retained. However, it remains formulated in terms of already-emergent physical and biomedical concepts: pressure, radius, volume, temperature, plasma state, membrane permeability, reactive species, tissue disruption, and radiation.

This motivates the relational–informational reinterpretation developed in the following sections. The standard hydrodynamic model provides the observable macroscopic dynamics of the bubble, while the relational framework assigns a deeper structural meaning to this dynamics. In that interpretation, the collapse or nonlinear oscillation of *R*(*t*) is not merely a reduction in geometric volume, but a rapid compression of relational accessibility; the rise in temperature, chemical activation, or mechanical stress corresponds to concentration of informational action density; and radiative or biological output corresponds to relaxation of a high-tension relational configuration into accessible relaxation channels.

Thus, the standard physical and biomedical picture is not rejected. It is used as the effective macroscopic layer on top of which the relational–informational interpretation is constructed.

## 3. Relational–Informational Reinterpretation

The standard physical and biomedical description of ultrasound-induced cavitation treats the bubble or microbubble as a hydrodynamic object embedded in a liquid, vascular, or soft biological medium and describes its dynamics through variables such as radius, pressure, gas temperature, ionization state, chemical activation, membrane permeability, tissue disruption, and, in sonoluminescent regimes, optical emission. In the relational–informational interpretation, these quantities are not rejected, but are supplemented by a model-level description in terms of constrained relational accessibility.

In the relational–informational framework, the underlying substrate is represented by a directed, weighted relational graph

(5) G=(V,E,C)

where *V* denotes a set of relational degrees of freedom, *E* ⊆ *V* × *V* denotes admissible informational relations between them, and

(6) $$C:E\to R_{\geq 0}$$

assigns a nonnegative constraint weight to each relation. The weight *C*(*e*) quantifies the cost, resistance, or limitation associated with relational information flow along edge *e*.

Effective distance is then defined by minimal accumulated constraint cost along admissible paths,

(7) $$d\left(i,j\right)=\underset{p:i\to j}{\inf}\sum_{e\in p}C(e)$$

so that geometry and locality emerge from constrained accessibility rather than from a primitive spatial manifold [15].

For ultrasound-induced cavitation, the bubble or microbubble region is modeled as a localized, time-dependent relational subgraph

(8) $$G_{b}(t)=(V_{b}(t),E_{b}(t),C_{b}(t))\subset G$$

whose effective boundary is represented macroscopically by the bubble radius *R*(*t*). In biomedical settings, this localized cavitation domain is coupled to an adjacent biological microenvironment, including membranes, extracellular matrix, vascular structures, molecular agents, or tissue interfaces. In the proposed interpretation, the acoustic field is not described only as acting on a pre-given geometrical cavity; rather, it is also represented as modulating the constraint structure *C_b_*(*t*) of this localized relational domain and its biological surroundings. Bubble oscillation or collapse therefore corresponds, in relational terms, to a rapid increase in constraint density and a reduction in the accessibility volume associated with *G_b_*(*t*).

The central idea is therefore that the acoustically driven bubble or microbubble acts as a localized relational domain in which constraints are dynamically intensified by external forcing. As collapse or nonlinear oscillation proceeds, the effective accessibility volume decreases, local constraint density increases, and the informational action density rises sharply. In sonoluminescence, the emitted light is interpreted as radiative relaxation of this highly constrained relational state into photon-like radiative motifs. In biomedical cavitation, the same high-tension state may relax through bioactive channels associated with membrane permeabilization, reactive oxygen species generation, molecular delivery, or mechanical tissue fragmentation.

To make the proposed reinterpretation explicit, Table 1 maps the principal variables of the standard cavitation description onto their relational–informational counterparts. The table does not replace the conventional hydrodynamic, thermodynamic, chemical, or biomechanical meaning of these variables; rather, it identifies the structural role each variable plays when the bubble is viewed as a localized relational domain whose accessibility structure is compressed by acoustic forcing.

Table 1 shows that the relational–informational interpretation preserves the standard observable variables while assigning them a deeper structural role. In this view, R(t) and $\dot{R}(t)$ are not discarded; they become the macroscopic traces of an evolving relational subgraph. Similarly, photon emission, membrane permeabilization, reactive oxygen species generation, molecular uptake, and tissue disruption are not denied, but reinterpreted as coarse-grained outputs of relaxation channels activated by a high-tension relational state. In the generalized biomedical formulation, these outputs are represented by channel-specific variables $B_{k}(t)$, while the sonoluminescent optical case remains the particular radiative channel $I_{\gamma }(t)$.

**Figure 1:**
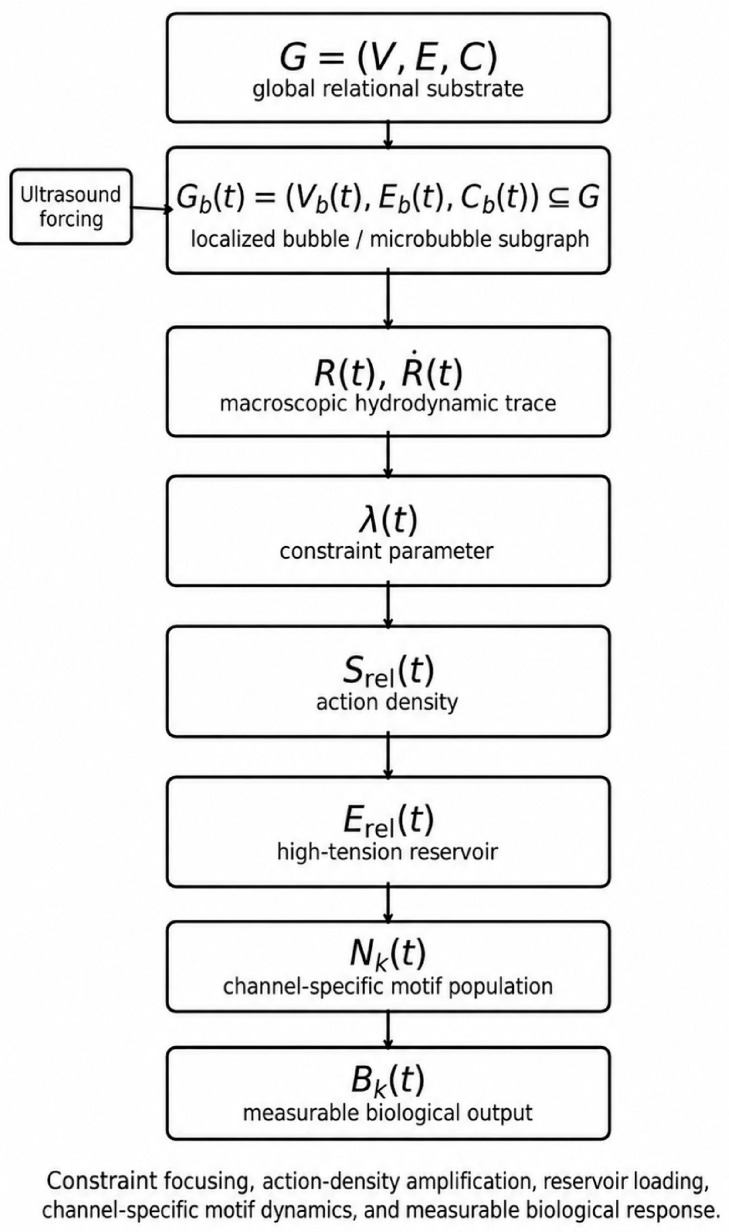
**Relational–informational bridge model for ultrasound-induced cavitation.** The global relational substrate is represented by a directed, weighted graph *G* = (*V*, *E*, *C*), where *V* denotes relational degrees of freedom, *E* admissible informational relations, and *C* constraint weights. The acoustically driven bubble or microbubble is modeled as a localized, time-dependent subgraph *G_b_*(*t*) = (*V_b_*(*t*), *E_b_*(*t*), *C_b_*(*t*)) ⊂ *G*. Its macroscopic hydrodynamic trace is described by the bubble radius *R*(*t*) and collapse or oscillation velocity $\dot{R}(t)$. These observable variables define a coarse-grained relational constraint parameter λ(t), which controls informational action density $S_{rel}(t)$ and loading of a stored high-tension reservoir $E_{rel}(t)$. Relaxation of this reservoir produces channel-specific motif populations $N_{k}(t)$, whose downstream expression appears as measurable biological outputs $B_{k}(t)$, including membrane permeability change, reactive oxygen species production, molecular uptake, tissue fragmentation, or, in the radiative sonoluminescent case, optical output.

This correspondence is summarized schematically in Figure 1. The figure emphasizes that the bridge model does not begin with the bubble radius alone, but with a localized relational subgraph *G_b_*(*t*) ⊂ *G*, whose evolving constraint structure is observed macroscopically through *R*(*t*) and $\dot{R}(t)$. In biomedical cavitation, *G_b_*(*t*) should be understood as coupled to a biological microenvironment that influences the available relaxation pathways.

This translation is grounded in the relational–informational framework, where the primitive structure is a network of degrees of freedom connected by admissible informational relations. In that framework, constraint weights are interpreted as path-additive costs that quantify the difficulty or resistance of relational information flow, while the informational action measures the global tension induced by these constraints.

Within this perspective, the bubble radius *R*(*t*) should not be regarded only as a geometrical variable. It is the emergent boundary of a localized relational domain. When *R*(*t*) is large or the bubble undergoes relatively stable oscillation, the internal degrees of freedom possess a relatively larger accessibility volume and lower effective constraint density. When *R*(*t*) rapidly decreases during collapse, the same relational subsystem is forced into a more restricted configuration space. The collapse therefore corresponds to a sharp reduction in relational accessibility.

The acoustic pressure field $P_{ac}(t)$ plays the role of an external control parameter. In standard physics, it drives the radial motion of the bubble. In the relational interpretation, it modulates the constraint structure of the localized domain. This is consistent with the framework’s definition of effective forces as responses to changes in coarse-grained constraint parameters. Within the relational–informational framework, effective forces may be represented as gradients of informational action or free energy with respect to parameters that modulate relational constraints.

Gas compression then becomes more than a thermodynamic event. It is the macroscopic expression of increasing local constraint density. As accessibility decreases, the relational degrees of freedom inside the cavitation domain become more tightly constrained. The corresponding rise in gas temperature is interpreted as a coarse-grained manifestation of increasing informational action density. In ordinary language, the gas becomes hot; in relational language, the localized subsystem enters a high-tension state.

Ionization, plasma-like behavior, chemical activation, or membrane-level perturbation can then be understood as the destabilization or reorganization of matter-like and bioactive relational motifs. In the relational–informational framework, particles and excitation-like entities are not primitive objects, but stable localized motifs of constrained information flow. Under extreme compression or nonlinear oscillation, these motifs may lose stability, reorganize, or open new relaxation channels. This provides a deeper structural interpretation of the transition from compressed gas to excited, chemically active, mechanically disruptive, or biologically responsive states.

Finally, the observable output is interpreted as relaxation into channel-specific motifs. In sonoluminescence, the light flash corresponds to the transition of the localized relational subsystem from a high-action, high-tension state toward a lower-tension configuration, with part of the released action appearing in the emergent electromagnetic description as photon emission. In biomedical cavitation, the corresponding relaxation may appear as membrane permeabilization, reactive oxygen species generation, molecular uptake, or tissue fragmentation. Symbolically, this may be written as

(9) $$G_{compressed}\to G_{relaxed}+H_{k}$$

where $G_{compressed}$ denotes the high-constraint relational configuration, $G_{relaxed}$ denotes the lower-tension post-collapse configuration, and $H_{k}$ denotes a channel-specific relaxation motif. The radiative sonoluminescent case corresponds to $H_{k}=H_{\gamma }$, whose emergent electromagnetic manifestation is the ordinary photon γ. In biomedical settings, $H_{k}$ may instead denote chemical, mechanical, permeability-related, or tissue-disruptive relaxation pathways.

Thus, in the relational–informational interpretation, ultrasound-induced cavitation is not simply a hydrodynamic collapse followed by thermal radiation or mechanical injury. It is a case of acoustic constraint focusing: an external acoustic drive compresses the accessibility structure of a localized relational domain until action density becomes sufficiently high to destabilize existing configurations and activate radiative or bioactive relaxation channels.

This interpretation preserves the standard physical and biomedical model while assigning it a deeper structural meaning. The Rayleigh–Plesset equation describes the observable effective dynamics of *R*(*t*), while the relational framework provides an additional structural interpretation of this dynamics in terms of constraint modulation, accessibility collapse, action-density amplification, motif destabilization, and radiative or bioactive release.

## 4. Minimal Bridge Model

To connect the standard physical and biomedical description of ultrasound-induced cavitation with the relational–informational interpretation, a minimal phenomenological bridge model is introduced. The purpose of this model is not to replace the Rayleigh–Plesset description of bubble dynamics, but to translate its central observable variable, the bubble or microbubble radius *R*(*t*), into a relational constraint parameter that measures acoustic constraint focusing.

The phenomenological quantities introduced below should be understood as coarse-grained summaries of the evolving cavitation subgraph

$$G_{b}(t)=(V_{b}(t),E_{b}(t),C_{b}(t))$$

In particular, the scalar parameter *λ*(*t*) summarizes the time-dependent constraint field *C_b_*(*t*), while $S_{rel}\left(t\right)$ represents the corresponding coarse-grained informational action density of the compressed relational domain. In biomedical settings, the same localized domain is coupled to an adjacent biological microenvironment, so that relaxation may occur not only through radiative channels, but also through membrane, chemical, molecular-delivery, or tissue-disruptive pathways.

The standard hydrodynamic model provides the time-dependent bubble radius *R*(*t*) and collapse or oscillation velocity $\dot{R}(t)$. In the relational–informational layer, these quantities are used to define a time-dependent constraint parameter λ(t), which represents the degree of relational compression inside *G_b_*(*t*). The following minimal form is proposed:

(10) $$\lambda \left(t\right)=\lambda_{0}+\alpha {\left(\frac{R_{0}}{R(t)}\right)}^{n}+\eta {\left(\tau_{c}\left|\frac{\dot{R}(t)}{R(t)}\right|\right)}^{m}$$

Here, $\lambda_{0}$ is the baseline constraint level, $R_{0}$ is a reference radius, α controls the contribution of geometric compression, η controls the contribution of collapse or oscillation rate and $\tau_{c}$ is a characteristic collapse time introduced so that the dynamical compression term is dimensionless. The exponents *n* and *m* are phenomenological parameters. The first nonlinear term expresses the fact that, as the bubble radius decreases, the internal relational domain becomes more constrained. The second nonlinear term expresses the fact that rapid collapse or strong nonlinear oscillation enhances constraint focusing beyond what is captured by radius alone. All terms in Equation (10) are dimensionless: $\frac{R_{0}}{R(t)}$ is dimensionless, and $\tau_{c}\left|\frac{\dot{R}(t)}{R(t)}\right|$ is dimensionless because $\frac{\dot{R}}{R}$ has units of inverse time. Thus, *λ*(*t*) is a dimensionless reconstructed compression-loading index. Thus, $\lambda \left(t\right)$ is a phenomenological dimensionless bridge variable, not a directly measured physical field. Its role is to summarize geometric compression and dynamic collapse or oscillation loading from R(t) and $\dot{R}(t)$, so that the subsequent variables $S_{rel}\left(t\right)$, $E_{rel}\left(t\right)$, $N_{k}\left(t\right)$, and $B_{k}\left(t\right)$ can be compared with endpoint-specific biological measurements.

This definition separates two physically important contributions. The factor

$${\left(\frac{R_{0}}{R(t)}\right)}^{n}$$

measures quasi-geometric compression: smaller bubble radius corresponds to reduced accessibility volume. The factor

$${\left(\tau_{c}\left|\frac{\dot{R}(t)}{R(t)}\right|\right)}^{m}$$

measures dynamical compression: faster collapse or stronger oscillation corresponds to stronger non-equilibrium focusing. This distinction is important because cavitation-mediated biological effects are not expected to depend only on acoustic pressure or minimum bubble radius, but also on the violence, rate, and temporal structure of the bubble dynamics.

The relational accessibility volume is then assumed to scale inversely with the constraint parameter:

(11) $$V_{acc}(t)\propto {\lambda (t)}^{-D_{eff}}$$

where $D_{eff}$ is an effective relational dimension. This expression means that increasing constraint strength reduces the number of relationally accessible configurations available to the localized cavitation subsystem. In the relational–informational framework, geometry is derived from constrained accessibility; therefore, a reduction in accessibility volume is the relational counterpart of bubble collapse or strong nonlinear cavitation focusing.

Next, a relational action density associated with the compressed cavitation state is defined. In the simplest phenomenological form,

(12) $$S_{rel}\left(t\right)=A{\lambda (t)}^{q}$$

where A>0 sets the scale of the action density and *q* > 0 controls the nonlinear amplification of action under constraint focusing. For *q* > 1, small increases in λ(t) near collapse can produce sharp action-density spikes, consistent with the temporal localization of sonoluminescent emission and with the threshold-like character of many cavitation-mediated biological effects. In the present phenomenological formulation, $S_{rel}\left(t\right)$ is treated as a normalized action-loading index. The scale factor *A* may be fixed by normalization in comparative studies or calibrated if an absolute endpoint scale is available.

More generally, the action density may be written as an integral over the localized cavitation domain:

(13) $$S_{rel}\left(t\right)=\int_{\Omega_{b}(t)}\Phi \left[C_{ij}(t)\right]d\mu$$

Here, $\Omega_{b}(t)$ denotes the relational domain associated with the bubble, $C_{ij}(t)$ are time-dependent relational constraint weights, Φ is a monotonic function of constraint strength, and μ is the measure over accessible relational degrees of freedom. This expression directly mirrors the general relational–informational construction in which the informational action quantifies the tension induced by constraints on relational information flow.

The observable output is then modeled as a relaxation process. A compressed, high-action relational configuration does not generate biological effect simply because $S_{rel}$ is large; it produces measurable output when relaxation channels become available. To express this distinction, a stored high-tension relational reservoir *E_rel_*(*t*) is introduced, loaded during rapid constraint focusing and depleted through radiative or bioactive relaxation channels:

(14) $$\frac{dE_{rel}}{dt}=\kappa_{load}{\left[\frac{dS_{rel}}{dt}\right]}_{+}-\kappa_{rel}(\lambda ,g,\chi_{bio})E_{rel}(t)$$

For an isolated collapse cycle, one may take $E_{rel}\left(t_{0}\right)=0$ before rapid compression begins, or treat it as a cycle-dependent residual reservoir in periodically driven steady states.

The notation ${\left[x\right]}_{+}$ denotes the positive part,

(15) $${\left[x\right]}_{+}=max(x,0)$$

The first term in Equation (14) loads the compressed relational state when the informational action density increases. The second term represents depletion of the high-tension reservoir through radiative, chemical, mechanical, permeability-related, and non-radiative relaxation channels. The function $\kappa_{rel}(\lambda ,g,\chi_{bio})$ is an effective relaxation rate depending on the constraint state λ, the gas or microbubble composition g, and the biological context $\chi_{bio}$, including tissue stiffness, membrane state, vascular confinement, oxygenation, sonosensitizer availability, or molecular cargo.

To represent the finite formation and observation time of channel-specific relaxation motifs, a coarse-grained motif population $N_{k}(t)$ is introduced. This variable is loaded by relaxation of the high-tension reservoir and depleted through downstream expression, escape, dissipation, or biological effect:

(16) $$\frac{dN_{k}}{dt}=\Gamma_{k}\left(\lambda ,g,\chi_{bio}\right)\kappa_{rel}\left(\lambda ,g,\chi_{bio}\right)E_{rel}\left(t\right)-\kappa_{out,k}N_{k}(t)$$

Here, $\Gamma_{k}\left(\lambda ,g,\chi_{bio}\right)$ is a channel-specific efficiency factor measuring the fraction of reservoir relaxation that couples to the *k*-th output pathway, while $\kappa_{out,k}$ controls the rate at which this motif population contributes to an observable output. In the radiative sonoluminescent case, *k* = *γ*, $N_{k}\left(t\right)=N_{\gamma }(t)$, and the output is optical. In biomedical cavitation, *k* may denote membrane permeabilization, reactive oxygen species generation, molecular uptake, or tissue fragmentation.

The measurable output associated with the *k*-th channel is then identified with the downstream expression of this motif population:

(17) $$B_{k}\left(t\right)=\kappa_{out,k}N_{k}(t)$$

For example, $B_{mem}\left(t\right)$ may represent membrane permeability change, $B_{ROS}\left(t\right)$ reactive oxygen species production, $B_{drug}\left(t\right)$ molecular or drug uptake, and $B_{frag}\left(t\right)$ tissue fragmentation. The sonoluminescent optical case remains the special radiative case:

$$I_{\gamma }\left(t\right)=\kappa_{esc}N_{\gamma }(t)$$

where $I_{\gamma }\left(t\right)$ is the observed optical intensity.

The bridge model therefore contains the following linked stages:

$$G=(V,E,C)\to G_{b}(t)\to \left\{R\left(t\right),\dot{R}\left(t\right)\right\}\to \lambda (t)\to S_{rel}(t)\to {E_{rel}\left(t\right)\to N}_{k}(t)\to B_{k}\left(t\right)$$

The first two levels define the relational substrate and its localized cavitation subdomain. The hydrodynamic variables $R\left(t\right)$ and $\dot{R}\left(t\right)$ provide the observable macroscopic trace of the evolving subgraph. The remaining levels describe constraint focusing, action-density amplification, high-tension reservoir loading, channel-specific motif formation, and measurable biological output. The symbol $N_{k}\left(t\right)$ denotes the coarse-grained population of channel-specific relational motifs $H_{k}$, while $B_{k}\left(t\right)$ denotes the effective biological output measured at the emergent biomedical level.

A threshold condition can also be introduced:

$$\lambda \left(t\right)>\lambda_{c}$$

or equivalently,

$$S_{rel}\left(t\right)>S_{c}$$

Below this threshold, cavitation or bubble oscillation may occur without significant biological effect. Above it, matter-like or bioactive relational motifs may become unstable, reorganized, or coupled to relaxation channels. In this form, the model naturally explains why not every cavitation event produces sonoporation, reactive oxygen species generation, molecular delivery, tissue fragmentation, or optical emission: strong acoustic exposure alone is insufficient unless the relational constraint state crosses the relevant threshold, loads the high-tension reservoir $E_{rel}\left(t\right)$, and couples efficiently to a channel-specific motif population $N_{k}\left(t\right)$.

The model also clarifies why the biological or optical output should occur near, but not necessarily exactly at, the instant of minimum radius or maximum acoustic pressure. In the present formulation, output is governed not by $S_{rel}\left(t\right)$ alone, nor simply by $-dS_{rel}/dt$, but by the relaxation of the stored high-tension reservoir $E_{rel}\left(t\right)$. Consequently, the maximum of $B_{k}\left(t\right)$ may be controlled by the combined timing of constraint loading, threshold crossing, reservoir buildup, channel-specific motif formation, and downstream expression. This provides a qualitative prediction: ultrasound-mediated cavitation effects should correlate not only with bubble compression or acoustic pressure, but also with the rate of constraint loading, the kinetics of reservoir relaxation, and the formation or expression dynamics of $N_{k}\left(t\right)$.

In this minimal bridge model, ordinary cavitation physics, biomedical ultrasound effects, and relational–informational physics are not competing descriptions. The Rayleigh–Plesset equation gives the effective macroscopic trajectory of the bubble or microbubble. The relational model interprets that trajectory as a time-dependent compression of accessibility, producing action-density amplification, loading of a high-tension reservoir, and subsequent relaxation into radiative or bioactive channels. The phenomenon is therefore reframed as

**bubble or microbubble dynamics → constraint focusing → action-density amplification → reservoir loading → channel-specific motif formation → measurable biological output**

This provides the mathematical core of the proposed interpretation. It allows ultrasound-induced cavitation to be treated not merely as a hydrodynamic or therapeutic phenomenon, but as a concrete biomedical example of acoustic constraint focusing in a localized relational domain, followed by the release of relational action into emergent biological outputs.

The variables introduced in the bridge model have different roles: some correspond to observable or simulated bubble-dynamical quantities, while others are latent relational variables introduced to describe constraint loading and radiative or bioactive relaxation. Table 2 summarizes these quantities and indicates how each one may be interpreted empirically or computationally. This table is intended to clarify which components of the model are directly measurable, which are reconstructed from R(t) and $\dot{R}(t)$, and which represent phenomenological bridge variables to be constrained in future numerical, experimental, or biomedical work.

Table 2 also clarifies the empirical status of the proposed model. The quantities R(t) and $\dot{R}(t)$ belong to the standard hydrodynamic layer and can be measured or simulated. The quantities λ(t) and $S_{rel}\left(t\right)$ are reconstructed bridge variables. The reservoir $E_{rel}\left(t\right)$, motif population $N_{k}\left(t\right)$, and output $B_{k}\left(t\right)$ describe the proposed storage-and-relaxation pathway leading to observable biological or optical effects. This separation is important because it makes the model testable: the predicted output functional can be compared against biological response data and against simpler predictors such as acoustic pressure, minimum radius $R_{min}$, or $\left|\dot{R}/R\right|$. The bridge variables introduced here should therefore be interpreted as latent phenomenological quantities. They are not proposed as independently measured physical fields, but as reconstructed variables derived from observable or simulated cavitation dynamics and fitted against endpoint-specific biological measurements. This distinction is essential for empirical use of the model: its value depends on whether these variables improve the organization of response timing, threshold behavior, or endpoint magnitude compared with conventional predictors alone.

To reduce post hoc flexibility, empirical applications of the model should separate fixed or reconstructed quantities from endpoint-fitted quantities. Acoustic exposure descriptors such as $P_{a}$, frequency, and mechanical-index-like quantities are measured or fixed. The bubble trajectory R(t), $\dot{R}(t)$ and $R_{min}$ are measured experimentally or obtained from a specified bubble-dynamical simulation. The bridge descriptors λ(t), $S_{rel}\left(t\right)$ and ${\left[{dS}_{rel}/dt\right]}_{+}$ are reconstructed from R(t) and $\dot{R}(t)$ after choosing the bridge exponents and normalization constants. In contrast, endpoint-specific quantities such as $\kappa_{rel}$, $\kappa_{out,k}$, and $\Gamma_{k}$ require endpoint data and should be estimated on training data and evaluated on held-out exposure conditions, microbubble formulations, or biological endpoints. The model would be weakened if acoustic pressure, $R_{min}$, mechanical index, $\left|\dot{R}/R\right|$, or cavitation-dose metrics consistently outperformed $B_{k}\left(t\right)$ under the same validation scheme.

Thus, the model does not require λ(t), $S_{rel}\left(t\right)$, *E_rel_*(*t*), or *N_k_*(*t*) to be independently observable microscopic fields. It requires that the endpoint functional derived from them, *B_k_*(*t*), be testable against measured or synthetic biological outputs and against conventional cavitation descriptors.

In the controlled synthetic benchmark reported below, the bridge-shape parameters were fixed before comparison, and the benchmark was used only to test temporal alignment and endpoint-magnitude organization against conventional descriptors, not to claim external biological validation.

## 5. Illustrative Numerical Examples and Controlled Synthetic Benchmark

### 5.1. Prescribed Collapse-Profile Illustration

To illustrate the qualitative behavior of the bridge model, consider a simplified collapse profile in which the bubble or microbubble radius reaches a minimum near *t* = *t*_c_:

$$R\left(t\right)=R_{min}+\left(R_{max}-R_{min}\right)\left[1-\exp\left(-\frac{{\left(t-t_{c}\right)}^{2}}{2\tau_{c}^{2}}\right)\right]$$

The symmetric form is chosen only to isolate the bridge-model response around a collapse minimum; realistic bubble trajectories are generally asymmetric and should be obtained from Rayleigh–Plesset-type dynamics in quantitative applications. This expression is not intended to replace standard bubble dynamics. It is used only as a controlled illustrative input that captures rapid contraction toward a minimum radius followed by re-expansion.

Substitution of $R\left(t\right)$ and $\dot{R}\left(t\right)$ into Equation (10) yields a time-dependent relational constraint parameter *λ*(*t*). The corresponding action density is then obtained from

$$S_{rel}\left(t\right)=A{\lambda (t)}^{q}$$

The stored high-tension reservoir evolves according to

$$\frac{dE_{rel}}{dt}=\kappa_{load}{\left[\frac{dS_{rel}}{dt}\right]}_{+}-\kappa_{rel}E_{rel}(t)$$

where $\kappa_{load}$ controls loading of the high-tension reservoir and $\kappa_{rel}$ controls reservoir relaxation. For the illustrative example, $\kappa_{rel}$ is treated as an effective constant, although in the full biomedical bridge model it may depend on the constraint state, gas or microbubble composition, and biological context.

To represent the finite formation and observation time of channel-specific relaxation motifs, a motif population $N_{k}(t)$ is introduced. This variable is loaded by relaxation of the high-tension reservoir and depleted through downstream biological expression, dissipation, or measurement:

$$\frac{dN_{k}}{dt}=\Gamma_{k}\kappa_{rel}E_{rel}\left(t\right)-\kappa_{out,k}N_{k}(t)$$

The measurable biological output is then written as

$$B_{k}\left(t\right)=\kappa_{out,k}N_{k}(t)$$

Here, $\Gamma_{k}$ controls the fraction of reservoir relaxation that enters the *k*-th output channel, while $\kappa_{out,k}$ controls the rate at which the channel-specific motif population contributes to the measured biological response. The index *k* may denote membrane permeabilization, reactive oxygen species generation, molecular uptake, tissue fragmentation, or, in the radiative sonoluminescent case, optical emission.

For example,

*k* = mem corresponds to membrane permeabilization or sonoporation;

*k* = ROS corresponds to reactive oxygen species generation;

*k* = drug corresponds to molecular or drug uptake;

*k* = frag corresponds to tissue fragmentation or histotripsy;

*k* = γ corresponds to the radiative sonoluminescent channel.

**Figure 2:**
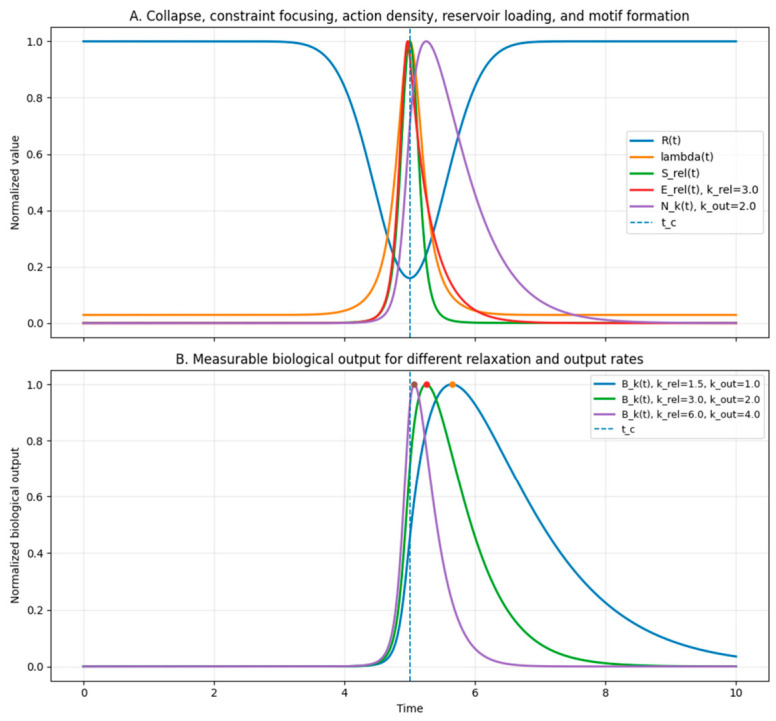
**Illustrative dynamical behavior of the relational–informational bridge model for a simplified cavitationprofile.** (**A**) The normalized bubble or microbubble radius R(t) reaches a minimum near $t_{c}$, while the relational constraint parameter *λ*(*t*) and informational action density $S_{rel}(t)$ increase during rapid compression. Positive action-density growth loads the high-tension reservoir $E_{rel}(t)$, which subsequently drives the formation of a channel-specific motif population $N_{k}(t)$. (**B**) The predicted measurable biological output $B_{k}(t)$ is shown for different relaxation and output rates. Slower relaxation or downstream expression produces delayed and broadened biological response, whereas faster relaxation and output produce a sharper response closer to the collapse time. The output maximum is therefore not determined by *R_min_* alone, but by the combined dynamics of constraint loading, reservoir depletion, and channel-specific motif formation. All curves are normalized for visual comparison.

The resulting normalized curves are shown in Figure 2. The figure is organized to separate the internal bridge-model mechanism from the predicted biomedical output. Panel A shows how collapse drives λ(t), $S_{rel}\left(t\right)$, $E_{rel}\left(t\right)$, and $N_{k}\left(t\right)$. Panel B shows how the measurable output $B_{k}\left(t\right)$ changes when the relaxation and output rates are varied. The example is not intended as a quantitative Rayleigh–Plesset simulation, but as a schematic dynamical demonstration of the proposed mechanism: geometric compression increases λ(t) and $S_{rel}\left(t\right)$, positive action-density growth loads $E_{rel}(t)$, reservoir relaxation produces channel-specific motifs $N_{k}(t)$, and their downstream expression generates the measurable biological output $B_{k}(t)$.

Figure 2 illustrates the central consequence of the bridge model: geometric collapse and biological output are dynamically separated. Although the bubble radius reaches its minimum near $t_{c}$, the measurable output $B_{k}(t)$ depends on the formation and downstream expression of channel-specific motifs from the stored high-tension reservoir. As a result, the biological response may occur near or after $t_{c}$, and its width depends on the relaxation and output rates. This supports the interpretation that ultrasound-mediated cavitation effects are governed not by minimum radius alone, but by the coupled process of constraint loading, reservoir relaxation, and channel-specific motif formation.

Figure 2 therefore provides a schematic illustration of the central claim of the bridge model: ultrasound-mediated cavitation effects are governed not by geometric collapse alone, but by the relaxation dynamics of a stored high-tension relational state into channel-specific bioactive motifs. The prescribed collapse profile is useful for isolating the internal logic of the model, but it remains an idealized input. To connect the bridge construction more directly with standard cavitation dynamics, the same equations were also implemented using a simplified Rayleigh–Plesset-type trajectory. This additional example is not intended as a patient-specific, tissue-specific, or experimentally fitted simulation, but as a minimal numerical demonstration that the proposed variables *λ*(*t*), $S_{rel}(t)$, $E_{rel}(t)$, and $B_{k}(t)$ can be reconstructed from conventional bubble dynamics.

In this Rayleigh–Plesset-driven implementation, the bubble radius R(t) was obtained by numerically integrating the simplified Rayleigh–Plesset equation introduced above, under sinusoidal acoustic forcing. The resulting R(t) and $\dot{R}(t)$ were then inserted into Equation (10) to reconstruct *λ*(*t*), followed by $S_{rel}(t)$, $E_{rel}(t)$, and $B_{k}(t)$ through Equations (12), (14) and (17). To avoid mixing several collapse–rebound events, the bridge variables were evaluated in a local time window centered on a dominant collapse. This local-window implementation resets the reservoir before the selected event and therefore isolates the sequence: geometric compression, dynamic loading, reservoir formation, and delayed endpoint expression.

### 5.2. Rayleigh–Plesset-Driven Local-Collapse Implementation

For the illustrative simulation, water-like parameters were used: ρ = 1000 kg m^−3^, μ = 10^−3^ Pa s, σ = 0.072 N m^−1^, P_0_ = 101,325 Pa, R_0_ = 2 μm, γ = 1.4, f = 1 MHz, and P_a_ = 0.9 × 10^5^ Pa. The bridge-model parameters were selected only to illustrate the delayed reservoir-mediated response and were not fitted to a specific experimental dataset.

These values were chosen as water-like, microbubble-scale, order-of-magnitude parameters for an illustrative cavitation calculation. The density, viscosity, and surface tension correspond to water-like media; R_0_ = 2 μm lies in the microbubble size range; *f* = 1 MHz is within the therapeutic-ultrasound frequency range; and P_a_ = 0.9 × 10^5^ Pa was selected to generate a clear local collapse without introducing unstable multi-collapse behavior. The bridge-model kinetic parameters were not fitted to biological data and were used only to illustrate reservoir-mediated delay.

**Figure 3:**
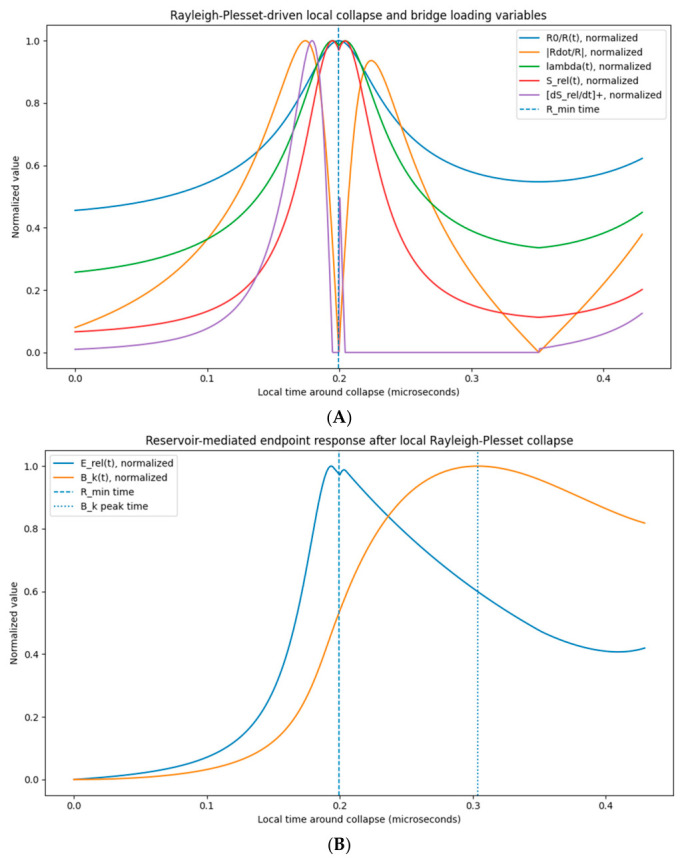
**Minimal Rayleigh–Plesset-driven implementation of the bridge model.** (**A**) A simplified Rayleigh–Plesset-type trajectory was used to generate a local collapse event. The resulting bubble trajectory was transformed into normalized compression and loading descriptors, including $R_{0}/R(t)$, $\left|\dot{R}/R\right|$, *λ*(*t*), $S_{rel}(t)$, and the positive loading term ${\left[{dS}_{rel}/dt\right]}_{+}$. The dashed vertical line indicates the time of minimum radius. (**B**) The reconstructed high-tension reservoir $E_{rel}(t)$, rises near the collapse event, whereas the predicted endpoint output $B_{k}(t)$ reaches its maximum after *R_min_*. This delayed peak illustrates that the model does not identify biological output with collapse severity alone, but with the sequence of dynamic loading, reservoir storage, relaxation, and downstream endpoint expression. Curves are normalized for visual comparison.

The Rayleigh–Plesset-driven example (see Figure 3) confirms the qualitative behavior observed in the prescribed-collapse demonstration. The minimum radius identifies the moment of strongest geometric compression, whereas the reservoir-mediated output reaches its maximum later, after the loading term has been transferred into $E_{rel}(t)$ and expressed through $B_{k}(t)$. Thus, even in a minimal Rayleigh–Plesset-type implementation, the predicted biological output is temporally separated from *R_min_*. This supports the use of $B_{k}(t)$, or its time-integrated value, as a candidate endpoint-specific predictor to be compared with acoustic pressure, mechanical index, *R_min_*, collapse velocity, or cavitation-dose metrics.

### 5.3. Controlled Synthetic Benchmark Against Conventional Predictors

To address how the proposed bridge output can be compared quantitatively with conventional cavitation descriptors, a controlled synthetic benchmark was added. This benchmark is not presented as biological validation. Rather, it is a reproducible positive-control numerical test designed to evaluate whether the endpoint functional $B_{k}(t)$ can recover a delayed response when compared with descriptors that do not include reservoir-mediated relaxation.

Sixty synthetic collapse events were generated by varying the minimum radius, maximum radius, collapse duration, collapse-center time, acoustic-pressure proxy, and frequency proxy. For each event, R(t) and $\dot{R}(t)$ were used to reconstruct λ(t), $S_{rel}(t)$, the positive loading term ${\left[{dS}_{rel}/dt\right]}_{+}$, the reservoir variable $E_{rel}(t)$, and the predicted endpoint functional $B_{k}(t)$. A synthetic delayed endpoint $Y_{k}(t)$ was then generated using the same storage–relaxation class of model but with slightly different hidden kinetic parameters, event-level amplitude modulation, and 5% additive noise. This avoided an exact identity between the candidate predictor and the synthetic endpoint.

The normalized endpoint trace $Y_{k}(t)$ was compared with $B_{k}(t)$, 1/R(t), $\left|\dot{R}/R\right|$, ${\left[{dS}_{rel}/dt\right]}_{+}$, and an acoustic-pressure trace. Temporal performance was quantified using root-mean-square error, peak-timing error, and Pearson correlation. In addition, endpoint magnitude was evaluated using five-fold cross-validated univariate linear regression for scalar predictors including $\int B_{k}(t)dt$, $maxB_{k}(t)$, $\int {\left[{dS}_{rel}/dt\right]}_{+}dt$, *R_min_*, 1/*R_min_*, $max\left|\dot{R}/R\right|$, acoustic-pressure proxy, and mechanical-index-like proxy.

**Figure 4:**
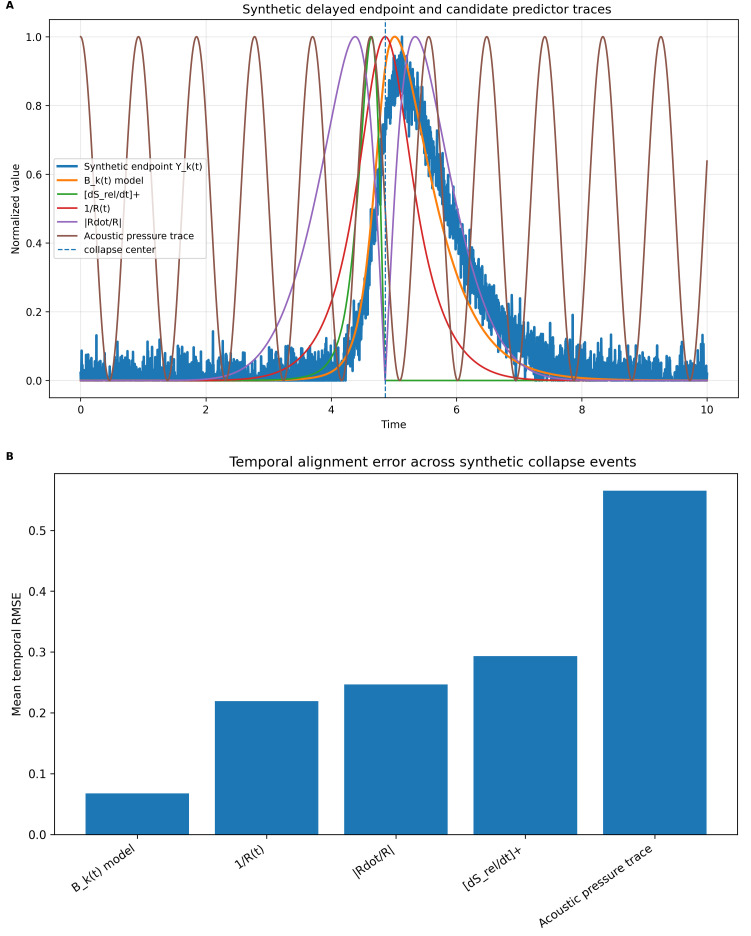
**Controlled synthetic benchmark comparing the bridge-model output with conventional cavitation descriptors.** (**A**) Representative synthetic delayed endpoint $Y_{k}(t)$ and candidate predictor traces, including $B_{k}(t)$, 1/R(t), $\left|\dot{R}/R\right|$, ${\left[{dS}_{rel}/dt\right]}_{+}$, and an acoustic-pressure trace. The benchmark is synthetic and is not intended as biological validation. It is used only to show how a delayed endpoint can be compared quantitatively with conventional descriptors. (**B**) Mean temporal root-mean-square error across 60 synthetic collapse events. The bridge-model endpoint functional $B_{k}(t)$ produced the lowest temporal error in this controlled delayed-response benchmark.

The results are shown in Figure 4 and summarized in Table 3 and Table 4. In this controlled delayed-response setting, $B_{k}(t)$ showed the lowest temporal error and the strongest correlation with the synthetic endpoint. This result should be interpreted narrowly: it does not validate the model against biological data, but it demonstrates a reproducible quantitative evaluation procedure and confirms that the bridge output can be compared directly to conventional descriptors under a delayed endpoint scenario.

## 6. Interpretation of Bioactive Cavitation Effects

The bridge model developed in the previous section suggests that the biological effects of ultrasound-induced cavitation should not be associated with geometric collapse alone. A small bubble or microbubble radius is an important condition because it amplifies compression and local constraint loading, but it is not sufficient by itself to account for membrane permeabilization, reactive oxygen species generation, molecular uptake, tissue fragmentation, or optical emission. In the present interpretation, measurable output occurs when the localized relational subsystem reaches a critical regime in which ordinary matter-like or bioactive configurations become unstable and relaxation into channel-specific motifs becomes accessible. The variable $N_{k}(t)$ introduced above represents the coarse-grained population of such $H_{k}$ -type motifs.

During the final stage of collapse, or during strong nonlinear oscillation, the bubble or microbubble region is conventionally described as a highly compressed and dynamically stressed domain, possibly reaching excited, chemically active, ionized, plasma-like, or mechanically disruptive states. In the relational–informational language, these descriptions correspond to a rapid reorganization of stable matter-like and bioactive motifs under extreme constraint focusing. The acoustic drive forces the localized domain into a state of reduced accessibility volume and increased constraint density. As a result, the system enters a high-tension relational configuration whose relaxation can appear, at the effective biomedical level, as membrane permeabilization, reactive oxygen species production, molecular delivery, tissue fragmentation, or, in the radiative case, photon emission.

This transition may be written schematically as

$$G_{compressed}\to G_{relaxed}+H_{k}$$

Here, $G_{compressed}$ denotes the high-constraint relational configuration generated near maximum collapse or strong nonlinear oscillation, while $G_{relaxed}$ denotes the lower-tension configuration reached after relaxation. The term $H_{k}$ denotes a channel-specific relaxation motif. In the radiative sonoluminescent case, $H_{k}=H_{\gamma }$, whose emergent electromagnetic manifestation is the ordinary photon *γ*. In biomedical cavitation, however, $H_{k}$ may denote a chemical, mechanical, permeability-related, or tissue-disruptive relaxation pathway.

This interpretation is consistent with the broader relational framework, in which particle-like and excitation-like entities are understood as stable localized motifs of constrained information flow, rather than as fundamental objects. Under ordinary conditions, atoms, molecules, membranes, extracellular structures, and electromagnetic excitations appear as stable effective structures. Under intense cavitation, however, the local constraint landscape changes abruptly. The stability of these configurations may be lost, reorganized, or coupled to new relaxation pathways. The observable biological effect is therefore interpreted as the coarse-grained trace of relational action released into channel-specific motifs.

This also clarifies why cavitation-mediated biological effects are temporally localized and threshold-dependent. The system does not necessarily produce biological response throughout the entire acoustic cycle. Significant output is expected only during the interval in which the compressed or strongly oscillating configuration both exceeds the relevant constraint threshold and can relax through an accessible biological or radiative channel. Consequently, the maximum biological output need not coincide exactly with the instant of minimum radius or maximum acoustic pressure. It may instead correspond to the time at which the compressed relational state releases action most efficiently into the relevant channel-specific motifs.

Gas composition, microbubble properties, and biological context acquire a natural structural role in this interpretation. Noble gases, water vapor, dissolved gases, microbubble shell composition, sonosensitizers, drug payloads, tissue stiffness, oxygenation, vascular confinement, and membrane state do not merely alter thermodynamic or biomechanical parameters. They also affect motif stability and the availability of radiative, chemical, mechanical, permeability-related, or non-radiative relaxation pathways. In standard biomedical language, these factors modify heat capacity, cavitation threshold, shell dynamics, local shear stress, chemical activation, reactive oxygen species production, drug release, and tissue susceptibility. In relational language, they modify constraint accessibility, motif stability, and channel-specific relaxation efficiency.

Thus, ultrasound-induced cavitation is interpreted as a threshold-driven relational transition:

**constraint focusing → motif destabilization → channel-specific motif formation → measurable biological output**

The key claim is therefore not that biological effect occurs simply because the bubble becomes small or because acoustic pressure is high. Rather, ultrasound-induced cavitation drives the localized relational domain into a high-action state from which relaxation into specific bioactive pathways becomes favorable. Sonoporation, reactive oxygen species generation, molecular uptake, tissue fragmentation, and sonoluminescent light emission may therefore be interpreted as different coarse-grained outputs of the same deeper process: constraint focusing followed by relational action release into accessible relaxation channels.

## 7. Predictions and Falsifiable Expectations

The relational–informational interpretation proposed here is not intended merely as a change in vocabulary. Its value depends on whether it generates expectations that can be compared, at least qualitatively, with observable features of ultrasound-induced cavitation and its biological consequences. The minimal bridge model developed above leads to several testable or falsifiable expectations concerning response timing, output magnitude, microbubble and gas dependence, tissue-context dependence, response duration, channel specificity, and threshold behavior.

First, the biological output should correlate more closely with relational constraint loading and reservoir relaxation than with acoustic pressure or minimum bubble radius alone. In standard descriptions, cavitation-mediated effects are often associated with strong bubble collapse, inertial cavitation, or high acoustic exposure. In the present framework, however, the relevant quantity is not only the geometrical compression of the bubble or microbubble, but the sequence by which relational action is accumulated, stored, and released. Since the constraint parameter λ(t) depends on both R(t) and $\dot{R}(t)$, the buildup of the high-tension relational reservoir is expected to be controlled by quantities such as

$$\left|\frac{d\lambda }{dt}\right|$$

and, more directly, by the loading term

$$\;{\left[\frac{dS_{rel}}{dt}\right]}_{+}$$

However, the measurable biological output is not predicted to follow this loading term instantaneously. In the reservoir formulation, the output is controlled by relaxation of the stored high-tension state into channel-specific motif populations:

$$B_{k}\left(t\right)=\kappa_{out,k}N_{k}(t)$$

Thus, a first falsifiable expectation is that the timing and strength of the biological response should correlate more strongly with the combined dynamics of constraint loading, reservoir buildup, channel-specific motif formation, and reservoir depletion than with *R_min_* or acoustic pressure alone. If accurate time-resolved measurements or simulations of bubble radius, cavitation activity, and biological response are available, the model predicts that the output maximum should align with the interval of strongest effective channel-specific relaxation, even if this is shifted relative to the instant of minimum radius.

Second, gas composition, microbubble properties, and biological context should affect the output not only through conventional mechanical or thermodynamic parameters, but also through the stability and accessibility of relational motifs. In standard biomedical ultrasound, gas core, shell composition, microbubble size, concentration, sonosensitizer availability, tissue stiffness, perfusion, oxygenation, and membrane state may strongly influence cavitation behavior and biological effect. In the present interpretation, this dependence is encoded in the channel-specific factors $\Gamma_{k}(\lambda ,g,\chi_{bio})$ and $\kappa_{rel}(\lambda ,g,\chi_{bio})$. Different gas, microbubble, and tissue conditions modify the internal organization of the compressed relational subsystem and change the probability that relaxation proceeds through membrane-permeabilizing, chemical, molecular-delivery, mechanical, or radiative channels. Therefore, these factors are not treated merely as passive modifiers of cavitation threshold, heat capacity, viscosity, shell dynamics, or tissue susceptibility, but as structural factors controlling motif stability, relaxation kinetics, and channel accessibility.

Third, the duration of the biological response should reflect relaxation-channel kinetics. In the bridge model, the measurable output $B_{k}\left(t\right)$ is not determined only by the duration of hydrodynamic collapse or acoustic exposure. It is also determined by the timescale over which the stored high-tension relational reservoir is depleted into channel-specific motifs and then expressed as a biological output. A rapidly available channel should produce a shorter and sharper response, while delayed relaxation, downstream biological processing, or competing non-productive channels should broaden, delay, or suppress the output. In this formulation, the effective response timescale is approximately controlled by

$$\tau_{eff}=F(\kappa_{rel}^{-1},\kappa_{out,k}^{-1})$$

where *F* denotes an effective timescale determined by both reservoir relaxation and downstream channel expression. This suggests that measured response durations should contain information not only about bubble dynamics, but also about the kinetics of relational action release and biological expression.

Fourth, different biological endpoints should track different constraint-release pathways. In conventional terms, sonoporation, reactive oxygen species generation, molecular uptake, and tissue fragmentation are usually treated as distinct mechanistic consequences of ultrasound-induced cavitation. In the relational interpretation, these are interpreted as different coarse-grained outputs of the same high-tension cavitation state, depending on which relaxation channel is accessible. The output profile should therefore be understood as a map of the pathways through which relational action is released. If microbubble composition, sonosensitizer presence, tissue type, or acoustic regime changes the dominant biological endpoint without a simple corresponding change in collapse strength alone, this would support the idea that output is controlled by relaxation-channel structure rather than by compression amplitude alone.

Fifth, the model predicts threshold behavior. Cavitation alone should not guarantee biological effect. A measurable output should occur only when the relational constraint parameter exceeds a critical value,

$$\;\lambda \left(t\right)>\lambda_{c}$$

or equivalently when the relational action density exceeds a critical threshold,

$$S_{rel}\left(t\right)>S_{c}$$

This provides a natural explanation for why some ultrasound exposures produce little or no biological effect, while others generate membrane permeabilization, reactive oxygen species production, molecular uptake, or tissue fragmentation. In the relational interpretation, the system must satisfy two conditions: it must reach a sufficiently high-action compressed state, and it must possess accessible relaxation channels. If either condition fails, cavitation may occur without significant bioactive motif formation.

In empirical applications, $\lambda_{c}$ and $S_{c}$ should not be treated as universal constants. They may be calibrated from no-effect/effect transition data by identifying the threshold that best separates exposures with no detectable endpoint response from exposures producing measurable membrane permeabilization, ROS generation, molecular uptake, or tissue fragmentation. Such thresholds should be estimated on training data and then tested on held-out exposure conditions or biological preparations.

These expectations can be summarized as follows:

**strong cavitation ≠ biological effect by itself**

Rather,

**biological output ≈ threshold crossing + reservoir loading + formation/expression of** $N_{k}\left(t\right)$

The most direct empirical test of the present model would be to combine time-resolved measurements or simulations of R(t) and $\dot{R}(t)$, passive cavitation detection or cavitation-dose metrics, and measurable biological endpoints [21,22,23,24]. From R(t) and $\dot{R}(t)$, one can reconstruct λ(t), $S_{rel}(t)$, the reservoir variable *E_rel_*(*t*), the channel-specific motif population *N*_k_(*t*), and the predicted output functional

$$\;B_{k}^{model}\left(t\right)=\kappa_{out,k}N_{k}(t)$$

The relational prediction is that the observed biological output should be better organized by this action-storage, motif-formation, and relaxation functional than by acoustic pressure, $R_{min}$, or $\left|\dot{R}/R\right|$ alone. Failure of this correlation would weaken the proposed bridge model, while successful correlation would support the interpretation of ultrasound-induced cavitation as acoustic constraint focusing followed by radiative or bioactive relational relaxation. In practice, this can be evaluated by comparing the temporal alignment and predictive error of candidate predictors such as *R_min_*, ∣dλ/dt∣, ${\left[dS_{rel}/dt\right]}_{+}$ and $B_{k}^{model}\left(t\right)=\kappa_{out,k}N_{k}(t)$.

The model would be supported if $B_{k}^{model}\left(t\right)$ organizes response timing or endpoint magnitude better than acoustic pressure, *R_min_*, mechanical-index-like exposure descriptors, or $\left|\frac{\dot{R}(t)}{R(t)}\right|$ alone; conversely, if conventional predictors consistently outperform the bridge variables, the proposed model would be weakened.

## 8. Discussion

The relational–informational interpretation developed in this paper does not deny the standard physical or biomedical description of ultrasound-induced cavitation. Nonlinear bubble dynamics, gas compression, thermodynamic excitation, possible ionization, chemical activation, mechanical stress, membrane perturbation, and tissue disruption remain essential components of the effective description. The Rayleigh–Plesset equation and its extensions describe the observable macroscopic evolution of the bubble or microbubble, while thermal, chemical, biomechanical, plasma, and biomedical ultrasound models describe the effective mechanisms by which radiative or biological outputs are produced.

The proposed bridge model should also be positioned relative to existing quantitative descriptions of cavitation-mediated bioeffects. Rate-process and thermal-dose-type models describe biological effect as an accumulated consequence of exposure intensity and duration. Pharmacokinetic or compartmental models describe delivery, exchange, retention, and clearance of therapeutic agents between biological compartments. Cavitation-dose models use acoustic emissions, cavitation activity, or related exposure descriptors as predictors of biological effect. The present bridge model does not replace these approaches. Its contribution is to introduce an intermediate latent-state layer that separates cavitation loading, transient reservoir storage, channel-specific relaxation, and endpoint expression. In this sense, the relational layer adds a state-space organization between measured or simulated bubble dynamics and delayed biological outputs, while preserving conventional hydrodynamic, sonochemical, pharmacokinetic, and cavitation-dose descriptions as effective empirical layers.

The aim of the present work is different. The empirical use of the bridge model does not require acceptance of the full relational–informational ontology; in the present bioengineering formulation, the relational terminology is used operationally to define latent variables for compression, loading, storage, relaxation, and endpoint expression. These ordinary variables and mechanisms can be supplemented by a relational–informational description that organizes them as part of a common constraint-loading and relaxation process. In this view, pressure, volume, temperature, plasma state, membrane permeability, reactive oxygen species generation, molecular uptake, tissue fragmentation, and photon emission are not abandoned, but reinterpreted as coarse-grained manifestations of constrained relational accessibility. The acoustic field modulates the constraint structure of a localized relational domain; bubble oscillation or collapse reduces its accessibility volume; compression amplifies action density; matter-like or bioactive motifs destabilize or reorganize; and relaxation into channel-specific motifs produces the observed biomedical or radiative output.

This interpretation gives ultrasound-induced cavitation a broader structural meaning. It is not merely a hydrodynamic process in which acoustic forcing produces bubble motion, nor only a therapeutic mechanism in which ultrasound generates tissue effects. It becomes a medically relevant case of a general mechanism:

**external drive → constraint focusing → localized high-action state → motif destabilization → bioactive relaxation**

Within the relational–informational framework, this sequence is significant because it illustrates how an experimentally accessible biomedical phenomenon may be described as a transition between relational configurations. The bubble or microbubble is not only a cavity or contrast agent in a liquid or tissue-like medium; it is an emergent boundary condition that focuses relational constraints. The measurable output is not merely a mechanical, chemical, or optical by-product; it is the coarse-grained signature of action release into channel-specific motifs *H*_k_.

The proposed model also clarifies the relation between standard and relational descriptions. Ordinary physics and biomedical ultrasound remain the effective languages in which experiments are measured and modeled. The relational–informational theory supplies a deeper structural interpretation of why such effective variables arise and how they are organized. In this sense, the two descriptions are not mutually exclusive. Hydrodynamics gives the macroscopic trajectory *R*(*t*), while the relational layer interprets this trajectory as a compression of accessibility. Thermodynamics and chemistry describe heating, excitation, and reactive species formation, while the relational layer interprets these processes as action-density concentration and channel activation. Biomechanics describes membrane deformation or tissue fragmentation, while the relational layer interprets these effects as bioactive relaxation of a high-tension relational state.

The distinctive contribution of the present framework is the introduction of an intermediate relational layer between cavitation dynamics and biological effect. This layer separates geometric compression, action-density amplification, high-tension reservoir loading, channel-specific motif formation, and measurable output $B_{k}(t)$. This separation is important because it prevents the biological effect from being identified too simply with acoustic pressure, minimum bubble radius, or collapse strength alone. Instead, the model emphasizes the full dynamical sequence by which constraint loading is stored and then released through accessible relaxation channels.

The empirical claim of the present work is not that cavitation proves the relational–informational ontology, but that the reduced bridge variables derived from this ontology may provide a useful state-space representation for organizing cavitation-mediated biological outputs.

The limitations of the present work should also be made explicit. The bridge model is phenomenological. It does not derive the Rayleigh–Plesset equation from relational first principles, nor does it calculate absolute biological effect size, reactive oxygen species yield, membrane permeability change, drug uptake, tissue fragmentation volume, photon yield, emission spectra, or pulse width from microscopic dynamics. The functions λ(t), $S_{rel}\left(t\right)$, $E_{rel}\left(t\right)$, $N_{k}\left(t\right)$, $B_{k}\left(t\right)$, $\kappa_{rel}\left(\lambda ,g,\chi_{bio}\right),\kappa_{out,k}$ and $\Gamma_{k}(\lambda ,g,\chi_{bio})$ are proposed as structural bridge quantities, not as fully derived laws. Future work should therefore develop numerical implementations in which realistic bubble or microbubble dynamics are coupled to the relational action-release model and compared with time-resolved cavitation and biomedical response data.

Despite these limitations, the framework provides a clear conceptual and mathematical direction. It suggests that ultrasound-induced cavitation should be studied not only as a process of compression, heating, chemical activation, or mechanical disruption, but also as a process of constraint modulation and action release. The key observable is not only how small the bubble becomes or how intense the acoustic exposure is, but how rapidly and efficiently the compressed relational state can relax into specific biological or radiative channels. This shift in emphasis may help organize response timing, microbubble dependence, tissue-context dependence, threshold behavior, and endpoint specificity within a unified structural framework.

Thus, the present paper should be understood as a bridge between established cavitation physics, biomedical ultrasound mechanisms, and a relational–informational foundation of physical and biological description. It does not replace ordinary physics or biomedical modeling; it proposes an additional phenomenological layer for organizing how cavitation dynamics may be linked to delayed and channel-specific biological outputs.

## 9. Conclusions

In this work, a relational–informational interpretation of ultrasound-induced cavitation as acoustic constraint focusing followed by radiative or bioactive relaxation was proposed. Starting from the standard physical picture of an acoustically driven bubble or microbubble undergoing nonlinear oscillation or collapse, a bridge model was introduced in which the bubble radius R(t) and collapse or oscillation velocity $\dot{R}(t)$ define a time-dependent relational constraint parameter λ(t). This parameter controls accessibility compression inside the localized cavitation domain and determines an informational action density $S_{rel}(t)$.

Within this interpretation, cavitation-mediated biological effect is not attributed merely to acoustic pressure, collapse intensity, or the smallness of the bubble at collapse. Rather, measurable output occurs when acoustic forcing drives the localized relational subsystem into a high-action state from which relaxation into accessible channels becomes possible. These channels are represented by motif populations $N_{k}(t)$, whose downstream expression produces biological outputs $B_{k}(t)$. Depending on the channel, these outputs may include membrane permeabilization, reactive oxygen species generation, molecular or drug uptake, tissue fragmentation, or, in the radiative sonoluminescent case, optical emission.

The proposed model leads to several qualitative expectations. The timing and magnitude of the biological output should correlate with the combined dynamics of constraint loading, reservoir buildup, channel-specific motif formation, downstream expression, and gas-, microbubble-, or tissue-dependent relaxation efficiency, not only with *R_min_* or acoustic pressure. Microbubble composition, tissue context, oxygenation, sonosensitizer availability, and molecular cargo should influence output by modifying motif stability and channel accessibility. Response duration should reflect the kinetics of relaxation channels, while different biological endpoints should correspond to different pathways of constraint release. These expectations make the framework open to future numerical, experimental, and biomedical testing.

Ultrasound-induced cavitation may therefore be viewed as a biomedical manifestation of a general principle: when relational accessibility is rapidly compressed by an external acoustic drive, local action density can rise sharply, destabilizing matter-like or bioactive configurations and opening relaxation channels. The acoustic bubble or microbubble is not merely a hydrodynamic cavity, but an emergent boundary condition that focuses relational constraints until radiative, chemical, mechanical, permeability-related, or tissue-disruptive relaxation becomes possible.

In this sense, therapeutic cavitation provides a concrete biomedical setting in which the relational–informational framework can be connected to observable phenomena. Ordinary hydrodynamics, thermodynamics, chemistry, biomechanics, and biomedical ultrasound remain the effective descriptive layers, while the deeper relational interpretation organizes them as successive manifestations of constraint focusing, motif destabilization, reservoir-mediated action release, and channel-specific biological output.

## Figures and Tables

**Table 1:** Standard cavitation variables and their relational–informational interpretation.

Standard Variable	Relational Meaning	Role in Bridge Model
R(t)	emergent boundary of relational cavitation domain	input variable
$\dot{R}(t)$	collapse- or oscillation-rate focusing	dynamic loading term
$P_{ac}(t)$	external acoustic constraint modulation	drive parameter
gas compression	constraint-density increase	contributes to λ(t)
temperature rise	action-density concentration	coarse-grained proxy
plasma/ excitation	motif destabilization	threshold regime
ROS generation	chemical relaxation channel	bioactive output $B_{ROS}(t)$
molecular uptake	accessibility-opening biological output	delivery output $B_{drug}(t)$
tissue fragmentation	structural relaxation/ disruption	histotripsy output $B_{frag}(t)$
photon emission	relaxation into photon-like radiative motifs $H_{\gamma }$	optical output $I_{\gamma }(t)$

**Table 2:** Bridge-model quantities and empirical interpretation.

Quantity/Parameter	Meaning in Bridge Model	Empirical/Computational Role
$P_{a},f,MI$	Acoustic exposure descriptors	Measured or fixed exposure inputs
R(t)	Bubble or microbubble radius	Measured or simulated bubble trajectory
$\dot{R}(t)$	Collapse or oscillation velocity	Derived from R(t)
$R_{min}$	Minimum radius during collapse	Conventional comparator predictor
$G_{b}(t)$	Localized relational cavitation subgraph	Conceptual representation of bubble/microbubble domain
$C_{b}(t)$	Time-dependent constraint field	Coarse-grained through λ(t)
*n*, m	Bridge-shape exponents	Fixed a priori or varied in sensitivity analysis
α,η	Compression/loading weights	Fixed or normalized before endpoint fitting
λ(t)	Relational compression parameter	Reconstructed from R(t)$\;\mathrm{and}\;\dot{R}(t)$
*q*, A	Action-density exponent and scale	Fixed or normalized before endpoint fitting
$S_{rel}\left(t\right)$	Informational action density	Reconstructed bridge-loading variable
${\left[{dS}_{rel}/dt\right]}_{+}$	Positive loading term	Reconstructed reservoir-input term
$\kappa_{load}$	Reservoir loading coefficient	Fixed or normalized in comparative tests
$E_{rel}\left(t\right)$	Stored high-tension reservoir	Latent state controlling delayed output
$\kappa_{rel}$	Reservoir relaxation rate	Endpoint-fitted or predefined kinetic parameter
$N_{k}(t)$	Channel-specific motif population	Intermediate latent channel variable
$\kappa_{out,k}$	Channel-output rate	Endpoint-fitted or predefined kinetic parameter
$\Gamma_{k}$	Channel-specific efficiency	Endpoint-, gas-, microbubble-, tissue-, or sonosensitizer-dependent fitted parameter
$B_{k}\left(t\right)$	Predicted endpoint functional	Model output compared with measured or synthetic endpoint
$\lambda_{c},S_{c}$	Threshold parameters	Calibrated from no-effect/effect transition data

**Table 3:** Temporal alignment of candidate predictors with the synthetic delayed endpoint.

Predictor	Temporal RMSE, Mean	Peak-Timing Error, Mean	Correlation, Mean
$B_{k}(t)$ model	0.0675	0.1313	0.9689
1/R(t)	0.2192	0.3589	0.5658
$\left|\dot{R}/R\right|$	0.2466	0.4313	0.5913
${\left[{dS}_{rel}/dt\right]}_{+}$	0.2933	0.5017	0.0712
Acoustic-pressure trace	0.5652	2.5524	−0.0431

Note: Values are averaged across 60 synthetic collapse events. The benchmark is a controlled synthetic positive-control test and should not be interpreted as biological validation.

**Table 4:** Five-fold cross-validated prediction of synthetic endpoint magnitude using scalar predictors.

Scalar Predictor	CV Normalized RMSE	CV R^2^
$maxB_{k}(t)$	0.0237	0.989
$\int {\left[{dS}_{rel}/dt\right]}_{+}dt$	0.0243	0.989
$\int B_{k}(t)dt$	0.0243	0.989
$max{\left[{dS}_{rel}/dt\right]}_{+}$	0.0682	0.911
1/*R_min_*	0.0830	0.868
*R_min_*	0.1267	0.693
$max\left|\dot{R}/R\right|$	0.2190	0.0838
MI-like proxy	0.2412	−0.112
Acoustic-pressure proxy	0.2454	−0.151

Note: Because the endpoint is synthetic and delayed by construction, these values demonstrate benchmark behavior and reproducibility, not external biological validation.

## Data Availability

No new experimental or clinical data were generated or analyzed in this theoretical study. The numerical scripts and parameter values used to generate Figure 2, Figure 3 and Figure 4 and Table 3 and Table 4 are provided as Supplementary Code S1–S3. The synthetic benchmark outputs are provided as Supplementary Tables S1 and S2.

## Supplementary Materials

The following supporting information can be downloaded at https://www.mdpi.com/article/10.3390/bioengineering13070832/s1, Code S1: prescribed collapse-profile bridge-model illustration used for Figure 2; Code S2: Rayleigh–Plesset-driven local-collapse implementation used for Figure 3; Code S3: controlled synthetic benchmark used for Figure 4 and Table 3 and Table 4; Table S1: temporal-alignment benchmark outputs; Table S2: scalar endpoint-prediction benchmark outputs.
