# Supplementary material for: Ultrasound-Induced Cavitation as Biological Constraint Focusing: A Phenomenological Bioengineering Model for Sonoporation, Sonodynamic Therapy, Drug Delivery, and Histotripsy
Source: Bioengineering (Basel). 2026 Jul 21;13(7):832. doi: 10.3390/bioengineering13070832 (PMC13406022; doi:10.3390/bioengineering13070832)
Supplement: Supplementary file 1 [file bioengineering-13-00832-s001.zip › Supplementary_Materials_bioengineering-4389138.pdf]

Supplementary Materials

Ultrasound-Induced Cavitation as Biological Constraint Focusing: A Phenomenological Bioengineering Model for Sonoporation, Sonodynamic Therapy, Drug Delivery, and Histotripsy

Supplementary Code S1-S3 and Supplementary Tables S1-S2

Supplementary material contents

Item	File name	Description
Code S1	Code_S1_prescribed_collapse_profile.py	Prescribed collapse-profile bridge-model illustration used for Figure 2.
Code S2	Code_S2_Rayleigh_Plesset_bridge.py	Rayleigh–Plesset-driven local-collapse implementation used for Figure 3
Code S3	Code_S3_controlled_synthetic_benchmark.py	Controlled synthetic benchmark used for Figure 4 and Tables 3 and 4
Table S1	TableS1_temporal_alignment_benchmark.csv	Temporal alignment benchmark comparing candidate predictor traces with the synthetic delayed endpoint.
Table S2	TableS2_scalar_endpoint_benchmark.csv	Five-fold cross-validated scalar endpoint-magnitude benchmark.

Reproducibility note. The scripts are provided as plain Python files. Code S3 uses a fixed random seed (4389138) for the controlled synthetic benchmark. The benchmark is a synthetic positive-control test and is not intended as biological validation.

Required Python packages. numpy, pandas, matplotlib, scipy, and scikit-learn. Code S1 uses numpy and matplotlib; Code S2 uses numpy, matplotlib, pandas, and scipy; Code S3 uses numpy, pandas, matplotlib, and scikit-learn.

Table S1. Temporal-alignment benchmark outputs

Temporal alignment of candidate predictor traces with the synthetic delayed endpoint. Values are averaged across 60 synthetic collapse events. The benchmark is a controlled synthetic positive-control test and should not be interpreted as biological validation.

Predictor	Temporal RMSE, mean	Temporal RMSE, SD	Peak-timing error, mean	Peak-timing error, SD	Correlation, mean	Correlation, SD
B_k(t) model	0.0675	0.0015	0.1313	0.0694	0.9689	0.0009
1/R(t)	0.2192	0.0281	0.3589	0.0906	0.5658	0.1325
|Rdot/R|	0.2466	0.0095	0.4313	0.3006	0.5913	0.0545
[dS_rel/dt]+	0.2933	0.0054	0.5017	0.0709	0.0712	0.0463
Acoustic pressure trace	0.5652	0.0090	2.5524	1.5615	-0.0431	0.0291

Table S2. Scalar endpoint-prediction benchmark outputs

Five-fold cross-validated prediction of synthetic endpoint magnitude using scalar predictors. Because the endpoint is synthetic and delayed by construction, these values demonstrate benchmark behavior and reproducibility, not external biological validation.

Scalar predictor	CV RMSE	CV normalized RMSE	CV R2
max B_k(t)	44.6498	0.0237	0.9892
int [dS_rel/dt]+ dt	45.7700	0.0243	0.9887
int B_k(t) dt	45.7801	0.0243	0.9887
max [dS_rel/dt]+	128.4	0.0682	0.9110
1/R_min	156.2	0.0830	0.8683
R_min	238.4	0.1267	0.6933
max |Rdot/R|	412	0.2190	0.0838
MI-like proxy	453.8	0.2412	-0.1120
Acoustic-pressure proxy	461.7	0.2454	-0.1510

Supplementary Code descriptions

Code S1. Code_S1_prescribed_collapse_profile.py

Prescribed collapse-profile bridge-model illustration used for Figure 2.

Main outputs: figure2_biomedical_bridge_model.png.

Main purpose: illustrate prescribed collapse, constraint focusing, reservoir loading, motif formation, and endpoint output for different relaxation/output rates.

Code S2. Code_S2_Rayleigh_Plesset_bridge.py

Rayleigh–Plesset-driven local-collapse implementation used for Figure 3.

Main outputs: Figure3A_RP_local_bridge_loading_clean.png, Figure3B_RP_local_bridge_output_clean.png, RP_bridge_predictors_clean_local_window.csv, and Table3_predictor_comparison_clean.csv.

Main purpose: integrate a simplified Rayleigh-Plesset equation, extract a local collapse window, and reconstruct bridge variables from R(t) and Rdot(t).

Code S3. Code_S3_controlled_synthetic_benchmark.py

Controlled synthetic benchmark used for Figure 4 and Tables 3 and 4.

Main outputs: Figure4A_synthetic_trace_example.png, Figure4B_temporal_RMSE_benchmark.png, TableS1_temporal_alignment_benchmark.csv, and TableS2_scalar_endpoint_benchmark.csv.

Main purpose: generate 60 controlled synthetic collapse events and compare B_k(t) against conventional descriptors.

Run instructions

Place each Python script in a writable working directory and run it with Python. For example:

```
python Code_S1_prescribed_collapse_profile.py

python Code_S2_Rayleigh_Plesset_bridge.py

python Code_S3_controlled_synthetic_benchmark.py
```

The CSV files included in this supplement are the benchmark outputs corresponding to Code S3.
